# Antigenic Variability a Potential Factor in Assessing Relationship Between Guillain Barré Syndrome and Influenza Vaccine – Up to Date Literature Review

**DOI:** 10.7759/cureus.10208

**Published:** 2020-09-02

**Authors:** Ravi Soni, Stacey E Heindl, Dwayne A Wiltshire, Ilmaben S Vahora, Safeera Khan

**Affiliations:** 1 Neurology, California Institute of Behavioral Neurosciences & Psychology, Fairfield, USA; 2 Medicine, California Institute of Behavioral Neurosciences & Psychology, Fairfield, USA; 3 Internal Medicine, California Institute of Behavioral Neurosciences & Psychology, Fairfield, USA

**Keywords:** guillain-barré syndrome, influenza vaccine, pandemic influenza vaccine, seasonal influenza vaccine, influenza-like illness, influenza, gbs

## Abstract

Guillain-Barré syndrome (GBS) is a possible serious adverse event of the influenza vaccine but it is yet to be proven. The objective of our traditional literature review is to assess the potential relationship between GBS and influenza vaccine. A traditional literature review has been carried out by selecting 26 articles from PubMed published between 2011 and 2020. Twenty-six articles met the selection criteria (eight observational studies, four systematic literature review, three meta-analyses, two case-control, two retrospective cohort, and seven case series). Selected studies were focused on monitoring the safety of influenza vaccines, the relative safety of pandemic and seasonal influenza vaccines, influenza vaccine a potential etiology of GBS, and pathogenesis of post-vaccination GBS. Few studies have shown a higher incidence of GBS with a pandemic influenza vaccine compared to the seasonal influenza vaccine, while several studies have concluded a small increase in the possibility of GBS following any type of influenza vaccine. There were some studies that estimated no association possibly due to the presence of confounding factors such as influenza-like illness, low power of the study, and reporting bias in post-vaccination surveillance programs. GSB should be taken into consideration as one of the less common but serious side effects of the influenza vaccine but it should not adversely affect the acceptance of the influenza vaccination program. Continuous monitoring of influenza vaccine safety should be performed regularly.

## Introduction and background

Guillain-Barré syndrome (GBS) is an autoimmune disorder affecting the peripheral nervous system presenting with acute onset flaccid polyneuropathy [1]. GBS is known to be the most common cause of acute flaccid paralysis worldwide [2]. The exact mechanism for developing GBS is largely unknown; however, the majority of cases are reported within two weeks after gastrointestinal or respiratory tract infection. The most frequent etiologies for the development of GBS are gastrointestinal infections, respiratory tract infection, surgery, and influenza vaccines. Pathogens include Cytomegalovirus, Campylobacter jejuni, Influenza, and Mycoplasma pneumoniae [3]. It is widely believed that GBS is an immune-mediated destruction of myelin sheaths of peripheral nerves in response to antibody development from bacterial or viral agents [3]. Fewer known risk factors are linked with the development of GBS, such as vaccination and surgery [2,3]. Intravenous immunoglobulin (IVIG) is a mainstay of treatment for GBS. According to the Center for Disease Control and Prevention (CDC), GBS affects 1 in 100,000 people with 3,000-6,000 new cases each year in the United States. GBS is highly prevalent among the male population and adults over the age of 50 [4].

Initial evidence of linkage between GBS and influenza vaccine was reported in the year of 1976, and the vaccination campaign was halted due to a higher incidence of GBS after rapid mass vaccination [5,6,7]. Since then, multiple studies have been conducted to define adverse events following the influenza vaccine. The link between GBS and influenza is a conflict of interest for many researchers; the potential association is yet to be clarified. After the 2009 pandemic of influenza A (H1N1) virus, mass vaccination campaigns have been initiated. To study the safety of the influenza vaccine, a surveillance program was started to monitor adverse events after vaccination [7]. This program has identified a small risk of GBS (incidence rate ratio 2·35, 95% CI 1·42-4·01, p=0·0003) following influenza vaccination in the years 2009-2010 [7]. Some studies have shown no association, while others have suggested a possible link with GBS after monovalent vaccine only in the years 2009-2010 [8].

From previously published articles and post-vaccine surveillance programs, there is a wide range of conflicting information available regarding GBS as a potential adverse event following influenza vaccination. Since the influenza vaccine is now recommended to everyone aged more than six months, it is essential to study the safety of the influenza vaccine. Post-vaccination surveillance programs are implemented to study the side effects of the influenza vaccine. Therefore, we have conducted a traditional literature review of post-vaccine surveillance data including published articles available from PubMed. The primary goal of our review is to identify influenza vaccine-related factors such as dose-dependent side effects, antigenic variability in vaccine component, and possible confounding factors that may play a role in causing GBS.

## Review

Method

An extensive literature search was performed using specific terms in PubMed to find related published articles. Keywords such as “Influenza vaccine, Flu vaccine, Guillain Barré syndrome and GBS” were used alone or in combination. Table 1 represents the results of the keywords used alone and in combination. One hundred twenty-six papers were extracted with the search term. We have used specific inclusion and exclusion criteria to finalize research papers relevant to our area of interest.

**Table 1:** Result by Keywords GBS: Guillain Barré syndrome

Keyword	Number of Articles in Pubmed
Influenza vaccine	33841
Flu vaccine	29834
Guillain Barré syndrome	10205
GBS	8313
Influenza vaccine & Guillain Barré syndrome	323
Influenza vaccine & GBS	126

Inclusion-Exclusion Criteria

We found 126 articles related to our topic. We included all types of studies conducted in the last ten years. Our studies included retrospective observational studies, case-control, retrospective cohort, case series, case report, systematic literature review, and meta-analyses from the post-vaccine surveillance data. Studies published in a language other than English were removed. Research studies involving animals were not included. We did not apply age-specific criteria as the goal of our study is to cover all age groups. We included studies from all over the world. Most of our studies are full-text articles, however, we did not exclude articles with only an abstract. With the application of inclusion and exclusion criteria, we shortlisted 26 studies out of 126 studies obtained through the search results.

Result

Out of 26 articles, eight articles were observational studies focusing on post-vaccine-surveillance data from the adverse event reporting system established by regulatory agencies [6,9-15]. There were four systematic literature reviews with one emphasizing the etiologies of GBS while three were focused on the association between influenza vaccine and development of GBS [2,3,5,16]. Three meta-analyses were included with an area of interest on the linkage of GBS with the specific type of influenza vaccine such as pandemic influenza vaccine and seasonal influenza vaccines [7,8,17]. There were two case-control and two retrospective cohort studies to estimate the risk of GBS after exposure to the influenza vaccine [18-21]. We included seven case series studies in which cases of GBS were identified by using specific diagnostic criteria and evaluated for previous exposure to the influenza vaccine [22-28]. The remaining three studies were used for basic understanding and comprehensive discussion on the pathophysiological aspect of GBS associated with the influenza vaccine [1,29,30]. Table 2 represents important studies from our review.

**Table 2:** Key studies selected in our review GBS: Guillain Barré syndrome

Author name	Publication Month-Year	Type of Study	Focus of study	Result / Conclusion
Sanz et al [5]	August 2019	Systematic literature review	Assess the risk of GBS after Pandemic & Seasonal influenza vaccine	GBS should be considered less common adverse event following influenza vaccine but it should affect vaccine acceptance.
Petras et al [8]	March 2020	Meta-analysis	Risk of GBS following seasonal influenza vaccine	The meta-analysis did not find association between GBS & seasonal influenza vaccine
Gattas et al [9]	December 2018	Retrospective observational	GBS & Seasonal trivalent influenza vaccine association	Post vaccination GBS is rare but should be monitored closely by health authorities
Bardenheier et al [11]	August 2018	Retrospective observational	Compare adverse event following pandemic and seasonal influenza vaccine in military vs civilian population aged 17-44 years	Incidence of GBS is four times higher in military population than civilian population.
Park et al [15]	July 2017	Retrospective Observational	Clinical and Laboratory features of GBS after influenza immunization	GBS should be suspected six weeks following immunization in the absence of clear alternative diagnosis to improve diagnostic accuracy and Brighton criteria is used for evaluation of post-vaccine GBS.
Dieleman et al [18]	July 2011	Case-control	Assess the risk of GBS after H1N1 pandemic influenza vaccine	No risk of GBS after the pandemic influenza vaccine when adjusted with influenza like illness and seasonal influenza vaccine.
Kuo et al [21]	July 2019	Case control	Association between GBS & Trivalent influenza vaccine in patient aged >50 years	Trivalent influenza vaccine has no association with GBS after 50 years of age
Prestel et al [27]	November 2014	Case series	Risk of GBS & variant Fisher syndrome following H1N1 pandemic influenza vaccine	High risk of GBS & Variant Fisher syndrome in temporal association with pandemic influenza vaccine

Discussion

Vaccines are known for preventing diseases. They are believed to be the greatest success in the field of medicine. Because of successful vaccination programs, the medical community has been focusing on adverse events of vaccines rather than disease prevention in recent years [9]. Several studies have been performed to investigate the safety of vaccines. Side effects of vaccines are a crucial factor for the acceptance of vaccination programs [30]. Influenza vaccines are not known to cause serious side effects [29]. However, since the 1976 origin of the swine influenza vaccine, various studies have been conducted worldwide to estimate the risk of GBS following different types of influenza vaccines. Post-vaccination GBS is evaluated by using the Brighton criteria [15]. Commonly studied vaccines are the monovalent pandemic influenza vaccine and the trivalent seasonal influenza vaccine. It is still controversial if there is an association between the influenza vaccine and GBS.

Molecular Mimicry - Possible Mechanism of Post-Vaccination GBS

The complete pathogenesis for the development of GBS is widely unknown. However, in the majority of the cases, immune-mediated damage of peripheral nerve cells following infectious or non-infectious processes leads to demyelination resulting in nerve damage [3]. GBS following vaccination is a rare occurrence. Antigenic challenge weeks prior to the onset of neurological symptoms have been identified in most of the cases of GBS [6]. Influenza vaccine may have antigenic cross-reactivity that stimulates antibody production against human neuronal cells. Antibodies formed with molecular mimicry attack human nerve cells because of structural similarity [16]. Anti-ganglioside antibodies damaging nerve cells are the known mechanism for the development of GBS [3,18]. Molecular mimicry with cross-reactivity to neuronal cells supports the causal relationship between the influenza vaccine and GBS [18].

Pandemic vs Seasonal Influenza Vaccine

During the influenza pandemic of 2009, the pandemic influenza vaccine was created by using the H1N1 strain of the influenza virus. Seasonal vaccines are available commonly every year with new formulation as the virus changes their genome [29]. Since the 2009 influenza pandemic, studies have been performed to compare the risk of GBS between the monovalent pandemic influenza vaccine and the trivalent seasonal influenza vaccine.

A study was conducted to assess the safety of the seasonal trivalent influenza vaccine by the pharmacovigilance department at Instituto Butantan in Brazil. They analyzed adverse event reporting following immunization from the years 2013 to 2017. There were seven cases of GBS reported during this time, supporting the relationship between GBS and the seasonal trivalent influenza vaccine. However, causality could not be determined because of a lack of sufficient medical information [9]. There was another noteworthy study in Korea, focusing on the clinical presentation of post-vaccination GBS provided comparative data on a specific type of influenza vaccine. As per claim-based data from 2002 to 2014, there were 48 cases of GBS. Out of 48 cases, 35 cases were due to the monovalent pandemic influenza vaccine, and 13 cases were from the trivalent seasonal influenza vaccine [15]. This study data indicated a higher risk of GBS after the monovalent pandemic influenza vaccine compared to the trivalent seasonal influenza vaccine.

A meta-analysis was performed by obtaining data from adverse event monitoring projects following the influenza A 2009 monovalent pandemic vaccination program in the USA, resulting in a slight increase risk of GBS (incidence rate ration 2.35, 95% CI 1.42-4.01, p=0.0003) [7]. There was a self-controlled case series supporting similar results estimating the relative incidence of GBS to be 2.42 (95% CI 1.58-3.72) following exposure to H1N1 pandemic influenza vaccine within 42 days with international collaboration [23]. Another meta-analysis provided the strength of association of GBS with pandemic vaccine and seasonal vaccine separately by investigating 39 studies. It discovered pandemic influenza vaccine has higher risk (relative risk = 1.84, 95% CI, 1.36-2.50) relative to seasonal influenza vaccine (relative risk 1.22, 95% CI, 1.01-1.48) [17]. Consistency in the above study results favors the association between GBS and the specific type of influenza vaccine.

A Norwegian population-based cohort study indicated that there is no association of GBS after exposure to the influenza A (H1N1) pandemic vaccine. However, the study provides crucial information on a higher risk of GBS following pandemic influenza infection [19]. Meta-analysis performed in the Czech Republic by Petras et al. was not able to establish the risk of GBS following inactivated trivalent seasonal influenza vaccine. On the other hand, their analysis explained that influenza and upper respiratory tract infection (influenza-like illness) have an elevated risk of GBS [8]. Another self-controlled case series in Europe provided a similar result. According to the study, the pandemic H1N1 influenza vaccine increased the risk of GBS when unadjusted with potential confounding. However, adjusted with confounding such as influenza-like illnesses did not find a notable association of GBS with pandemic H1N1 influenza vaccine [26]. In Germany, the case series data provided vital information on confounding factors with a potential relationship. The data indicated higher chances of GBS with the pandemic influenza vaccine, and a statistical analysis from the study showed that the presence of confounding did not have a significant effect on the final study result [27].

A recent article published in December 2019 by Arefeh et al. included 39 studies to monitor the possibility of GBS after receiving the pandemic and the seasonal influenza vaccine. An overview of these studies reported a slightly higher risk of GBS with the pandemic influenza vaccine in comparison to the seasonal influenza vaccine [29]. In Taiwan, a population-based case-control was conducted to determine the potential relationship between the seasonal trivalent influenza vaccine and GBS in hospitalized patients age >50 from 2007 to 2015. The study outcome did not prove the link between GBS and the seasonal trivalent influenza vaccine [21].

The above studies indicate that efforts had been made worldwide in several countries on GBS association with a specific type of influenza vaccine to assess the relative safety. However, the study results are inconsistent.

Post-Vaccination Surveillance

Following the 2009 pandemic of the H1N1 influenza infection, many countries have adopted a post-vaccine monitoring system for adverse events of the influenza vaccine. To keep track of post-licensure safety of the influenza vaccine, the Vaccine Adverse Event Reporting System (VAERS) was implemented by the Center for Disease Control and Prevention (CDC) and Food and Drug Administration (FDA) in the United States. Vaccine-related side effects must be reported to VAERS by healthcare providers for all licensed vaccines in the United States. After the launch of the 2009 pandemic influenza vaccination program, reporting to VAERS became simpler by giving contact details on record cards of influenza vaccine [11].

A study comparing military vs civilian populations that was conducted using post-vaccine surveillance data showed a high rate in the military population in the United States. The incidence of GBS was four times greater in military personnel than the civilian population aged 17-44 years. This difference could be due to strict reporting practices in military personnel [11]. Another relevant surveillance data analyzed by Sandhu et al. focusing on the Medicare population concluded an elevated risk in the years 2010-2011 but no risk in subsequent years with the same type of vaccine [10].

Active surveillance conducted by the National Center for Epidemiology in Spain evaluated the chances of GBS following the 2009 pandemic influenza vaccine and seasonal influenza vaccine from 2009 to 20011. Their analysis resulted in no association of GBS with both influenza vaccine likely due to under-reporting by neurologist network [22]. To determine the safety of the inactivated quadrivalent influenza vaccine, post-licensure surveillance was monitored using VAERS data from July 2013 to May 2015. They did not report serious side effects including GBS [12]. A systemic literature review conducted in the United States to find out the effect of power on study results found no association when they did not cover a large group of population. In contrast, studies involving different designs and different population with international collaboration showed evidence of higher risk of GBS following the influenza vaccine [2]. Another systemic literature review from three epidemiological studies in Spain estimated a small risk of GBS, but confounding makes it challenging to interpret the potential relationship between GBS and influenza vaccine [5].

Outcomes reported by the Canadian Pediatric Surveillance program and the Canadian Immunization Monitoring program actively emphasizing the pediatric population determined that post-immunization GBS is a less common but serious side effect in children [24]. A study published in the American Journal of Epidemiology concluded the risk of GBS following the 2009 H1N1 influenza vaccine among the Medicare population, but the risk was significantly lower than the 1976 swine influenza vaccine [28]. In Korea, medical records were reviewed for all GBS cases from 2008 to 2010 to estimate risk during the pandemic period. They observed a smaller risk of GBS following the pandemic influenza vaccine during the pandemic period; nevertheless, vaccination should not be stopped based on GBS as a serious side effect [25]. A population-based cohort study in Canada from October 2009 to March 2010 observed two cases per 1 million doses. They reported a smaller risk of GBS following the 2009 influenza vaccine in Quebec, Canada [20]. A surveillance statistics of Medicare beneficiaries from the year 2015 to 2017 concluded higher risk GBS following 8-21 days of vaccination, and high-dose influenza vaccine compare to standard dose [13]. Similar surveillance data in the year 2017-2018 analyzed by Perez et al. estimated no risk [14].

**Figure 1:**
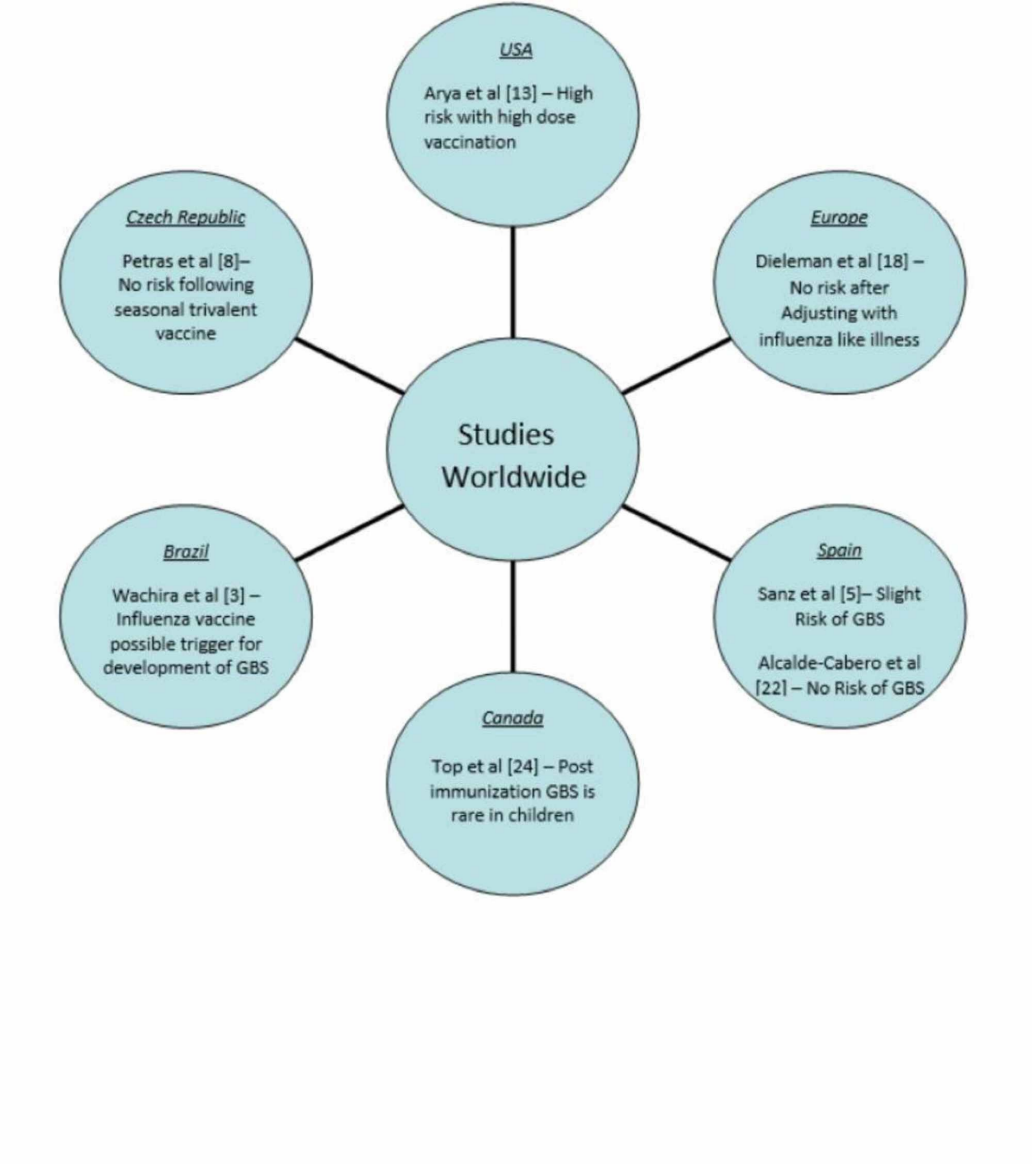
Important studies from all over the world GBS: Guillain Barré syndrome

Study results vary as it is difficult to draw conclusions based on established factors such as the presence of confounding, the specific type of influenza vaccine, diagnosing days following post-vaccination, and under-reporting to vaccine the surveillance program. Figure 1 represents important studies from all over the world.

Limitation

Our study has some limitations. We did not include studies older than 10 years or animal studies. We have excluded studies not published in the English language. Several studies are post-vaccination surveillance on adverse events following vaccination programs, there is the possibility of reporting bias. We were not able to find experimental studies on the safety of the influenza vaccine. There were a limited number of case reporting in recent years.

## Conclusions

GBS is a possible severe side effect of the influenza vaccine. Antigenic cross-reactivity is possible mechanism for post-vaccination GBS. Further studies should be conducted to evaluate antibody formation against specific strains of the influenza vaccine. The high-dose vaccine formula has shown a greater risk of GBS, which formulates the basis of studies to be conducted on the dose-dependent side effect of the influenza vaccine. Studies have been performed over the years to determine the safety of the influenza vaccine with post-licensure surveillance programs. Under-reporting was one the possible reason for not being able to detect a significant association between GBS and the influenza vaccine. Medical professionals working with the immunization group need to be trained in an accurate reporting system. Brighton criteria are used to diagnose post-vaccination GBS. A medical professional should suspect vaccine-related GBS in the absence of clear identified etiology. Despite the serious side effect as GBS, the weightage of the influenza vaccine is always high due to a beneficial effect in preventing severe complications of influenza.
